# Prediction and classification of chemical composition of ancient glass objects based on generalized Shapley functions

**DOI:** 10.3389/fchem.2024.1351143

**Published:** 2024-05-02

**Authors:** Na-Na Cai, Yi-Yuan Yin, Qi Han

**Affiliations:** ^1^ Shandong University of Science and Technology, Jinan, China; ^2^ Dean’s Office of Shandong University of Finance and Economics, Jinan, China

**Keywords:** ancient glass, gray prediction, generalized Shapley function, correlations, variability

## Abstract

Ancient glass products have suffered from the baptism of time and experienced changes in the burial environment and weathering, resulting in a change in the proportions of their chemical composition and interfering with their accurate identification by later generations. In this paper, the chemical composition of ancient glass products is predicted and identified. First, the multivariate statistical ANOVA test is applied to explore the relationship between whether the cultural relics samples are weathered or not and the glass type, decoration, and color to derive a law of chemical composition of the cultural relics and to analyze the correlation and difference among the four factors. Second, compared with the relevant data of the existing glass products, the missing values are processed by using the method of filling in the plurality. The weathering condition of the sampling points of the samples whose surfaces are not weathered is judged by the “distance discrimination method.” Combined with the characteristics of the lead-barium glass and the high-potassium glass, the law of the chemical composition content on the surface of the samples, weathered or not, is explored. The modeling of the gray prediction method was applied again to predict the chemical composition content before weathering. Finally, the generalized Shapley function of fuzzy measurement was used to analyze the correlation between indicators and the chemical compositions and their differences. The scheme proposed in this paper can solve the difficult problem of category judgment in archeology, which is of great significance in promoting the smooth progress of archaeological work.

## 1 Introduction

The identification of the production age of ancient glass products is one of the research topics in the field of archeology. Scientists determine the age of ancient glass products by analyzing and identifying their composition. However, the difference in chemical composition and quantity ratio during the glass production process leads to differences in its main chemical composition. For example, lead-barium glass contains high levels of lead oxide (lead monoxide) and barium oxide (barium oxide) due to the addition of a certain amount of lead ore; potassium glass contains substances with high potassium content (such as grass ash). The ancient glass had a certain degree of weathering before excavation, and due to the influence of the burial environment, its composition ratio changed, which, in turn, affected the correct judgment of its category by future generations. Therefore, understanding the chemical composition of ancient glass products has become an important factor in determining their classification.

In this paper, we analyze the relationship between the degree of weathering on the surface of cultural relics and the three aspects of glass type, ornamentation, and color for the available data related to glass products. We combine the characteristics of lead-barium glass and high-potassium glass to explore the pattern of chemical composition content on the surface of the samples with or without weathering. Finally, based on the given data, the chemical composition content before weathering is predicted. The generalized Shapley function of fuzzy measurement is applied to analyze the correlations between indicators and chemical compositions and to compare the differences in the correlations between the chemical compositions of the different categories. This paper provides a solution to the difficulty of judging categories in archaeological work, which is of great significance to the smooth progress of archaeological work.

## 2 Literature review

The current methods regarding the compositional exploration of glass artifacts are divided into two main categories: first, predictive analysis based on the chemical composition of glass, viscosity, temperature, fusion, surface weathering, and other data, and second, compositional exploration based on mathematical statistics.

Heredia-Langner et al. (2022) presented a model based on 3,935 viscosity-temperature data for 574 types of glass, aiming to predict the melting of alkaline-aluminum-viscous borosilicate glasses as a function of composition and temperature. Panova et al. (2021) described the three main types of Bohemian historical glass melting processes, illustrating the complexity of historical glass melting, as well as technological advances between periods. Liu et al. (2019) summarized the glass compositional system and production process of glass bead ornaments unearthed from the Han tombs in Lingnan and explored the provenance sources of the glass bead ornaments based on the chemical compositions and the style of the vessels.

Zhang et al. (2022) introduced seven simulation methods for glass component properties such as the summation method, the phase diagram method, Priven’s method, topological bound theory, molecular dynamics simulation, and mathematical-statistical simulation and summarized the main theoretical basis of each simulation method, the simulation process and the current status of its application. Alex (2004) investigated a method based on thermodynamic equations using the calculation program of MDL SciGlass and its database and using experimental data instead of some of the parameters of thermodynamic equations. Xu et al. (2021) vitrified low-activity waste (LAW) and high-level waste (HLW) and utilized the power of artificial intelligence to address the limitations of the existing models. Sharma et al. (2022) analyzed 25 “received” windshield samples from six automobile manufacturers for glass origin or complete chemical characterization using K-mean, cluster analysis, and principal component analysis (PCA) methods. Wang et al. researched the conservation and management strategies of cultural relics in archaeological excavations (Wang and Zheng, 2022).

Yang et al. (2022) proposed a feature study of glass artifacts based on BiLSTM-CRF. Lu (2023) integrated feature selection methods and classification algorithms in machine learning into the study of ancient glass artifact composition analysis and category identification problems and attempted to construct an integrated feature selection model for the chemical composition selection of ancient glass artifacts and a random forest model for identification and classification, using accuracy and AUC as the classification performance metrics. Kumiko et al. (2012) applied multiple regression analysis to predict glass structure. Melcher and Schreiner (2004) constructed two potassium-lime silica model glasses with compositions similar to medieval stained glasses. Qu et al. (2018) investigated some new generalized dual hesitant fuzzy generalized Choquet integral operators based on Shapley fuzzy measures.

Zou (2023) used time series and clustering methods for molecular compositional analysis of glass chemical compositions. Ye et al. (2015) investigated new gray prediction models to predict four typical interval gray number sequences separately. Zeng et al. (2014) developed a new interval gray number prediction model using kernel and area sequences of gray number bands. Meng et al. (2014) defined two generalized hesitant fuzzy Shapley–Choquet integral operators that globally consider the importance of the elements in a set as well as the correlation between them. Liu et al. (2022) investigated the topological properties of benzenoid planar octahedron networks.

## 3 Data sources and assumptions

The data in this paper come from Question C of the 2022 China University Student Mathematical Modeling Competition. Archaeologists categorized a batch of ancient glassware into two types, lead-barium glass and high-potassium glass, and measured the proportions of the main chemical components. The specific data are shown in Table 1. If the cumulative sums of the proportions of the components were not equal to 100% due to the means of detection and other reasons, any data with the cumulative sum between 85% and 105% were regarded as valid data. In order to facilitate the study of the problem, the following assumptions are made: 1) All data sources are authentic and reliable. 2) The steps of data cleaning and data preprocessing are accurate; that is, they are able to exclude outliers. Missing values of the chemical components that were not detected in the data were filled in with a value of 0, which had no effect on the rest of the data. 3) Some of the undetected trace data had no effect on the results. 4) Archeologists judged the known glass categories through conditions such as glass ornamentation and color.

**TABLE 1:** Selected data to be explored.

AN	Ornamentation	Typology	Color	Surface weathering	AN	Ornamentation	Typology	Color	Surface weathering
1	C	High potassium	Blue-green	Unweathered	11	C	Lead-barium	Pale blue	Weathered
2	A	Lead-barium	Pale blue	Weathered	12	B	High potassium	Blue-green	Weathered
3	A	High potassium	Blue-green	Unweathered	13	C	High potassium	Pale blue	Unweathered
4	A	High potassium	Blue-green	Unweathered	14	C	High potassium	Dark green	Unweathered
5	A	High potassium	Blue-green	Unweathered	15	C	High potassium	Pale blue	Unweathered
6	A	High potassium	Blue-green	Unweathered	16	C	High potassium	Pale blue	Unweathered
7	B	High potassium	Blue-green	Weathered	17	C	High potassium	Pale blue	Unweathered
8	C	Lead-barium	Purple	Weathered	18	A	High potassium	Deep blue	Unweathered
9	B	High potassium	Blue-green	Weathered	19	A	Lead-barium	-	Weathered
10	B	High potassium	Blue-green	Weathered	20	A	Lead-barium	Pale blue	Unweathered

*Where AN is the cultural relic number.

## 4 Research ideas

In this paper, the missing values are processed by the plurality filling method, the weathering conditions of the sampling points of the samples with unweathered surfaces are judged by the “distance discrimination method,” and the relationship between the weathering and the glass types, decorations, and colors are analyzed. It is hypothesized that the decoration of glass, as a symbol of regional culture, represents the geographical environment of different regions, and the natural environment of different regions is an important factor affecting the weathering results. When analyzing the relationship between glass type and weathering, this article takes ornamentation as a control variable. When analyzing the relationship between ornamentation and weathering, the special Type B ornamentation is analyzed first.

For the statistical analysis of the chemical composition content, this paper is divided into two parts of the study: high-potassium glass and lead-barium glass.

R software was used to test the gray prediction model addressing the chemical composition content of cultural relics before weathering, which includes the examination and processing of data, the establishment of the model, the examination of the predicted values, and the prediction of the forecast. The prediction model was solved, and the data were analyzed by MATLAB. The generalized Shapley function based on fuzzy measures analyzes the correlation relationship between indicators, analyzes the correlation relationship between their chemical compositions, and compares the differences in the correlation relationship of chemical compositions between different categories.

## 5 Exploring the characteristics of cultural relics

### 5.1 Data processing

#### 5.1.1 Missing color fill—plurality fill

**FIGURE 1:**
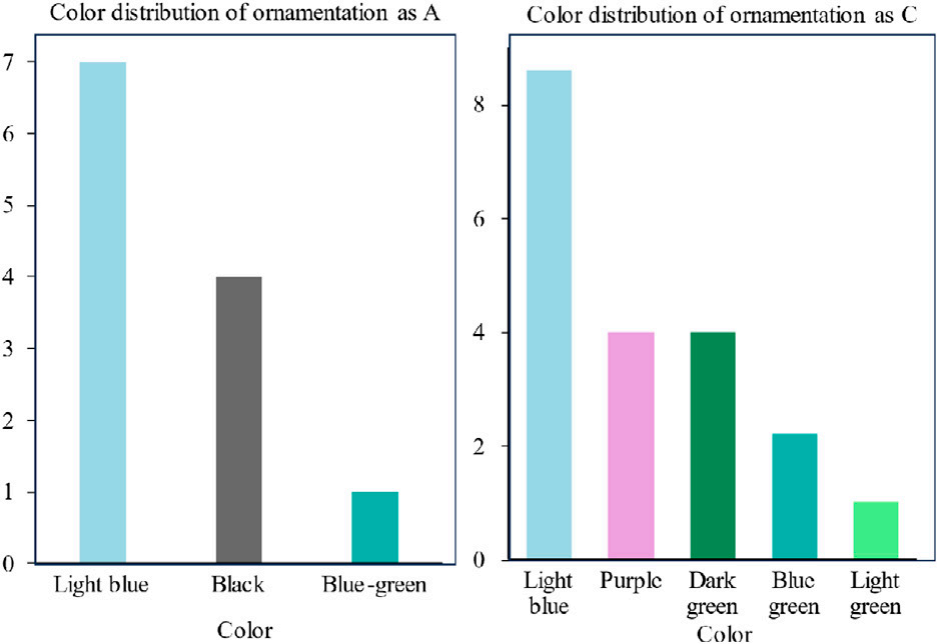
Color distribution of ornamentation as A and C.

Some values are missing from the color column in the given data. This paper uses the plurality filling method to deal with the missing values (Antti et al., 2010; Deng et al., 2019; Chen et al., 2020; Wang et al., 2022). Through reviewing the literature, it is known that the decoration of ancient glass is closely related to its use, and the color of cultural relics of the same use is also generally similar. In this paper, color data were missing for artifacts with ornamentation Types A and C. The statistics of these two types of decoration were used to obtain “pale blue” for the color of the mode. The color distributions of Type A and Type C decoration are shown in Figure 1.

#### 5.1.2 Outlier identification and handling—distance discriminant method

**FIGURE 2:**
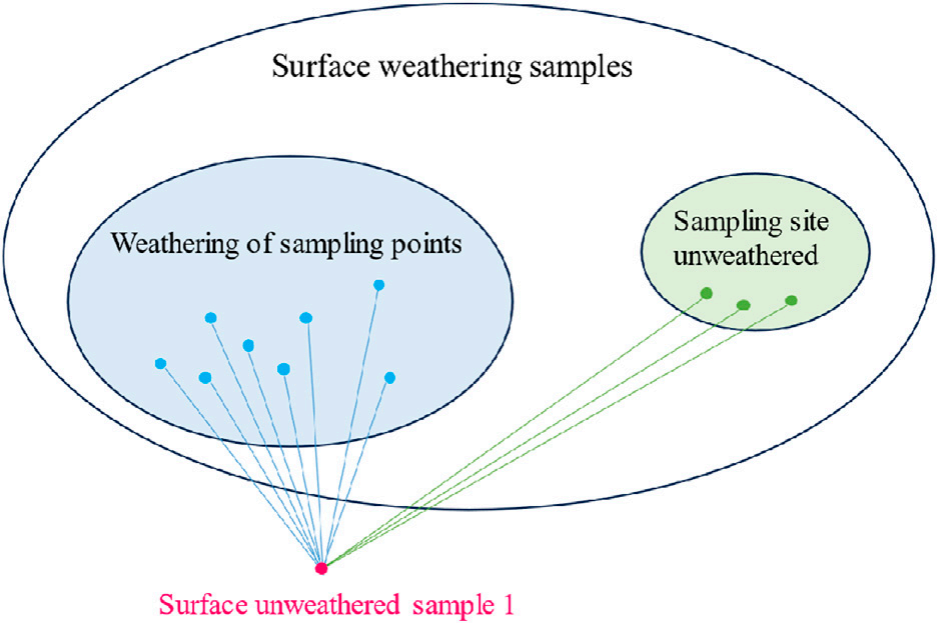
Schematic diagram of distance discrimination method.

In the following, the weathering condition of the sampling points on the surface of the unweathered samples was judged by the distance discrimination method (Gong and Lu, 2014; Flamary et al., 2018; Ye et al., 2018). A schematic diagram of the distance discrimination method is shown in Figure 2.

$$D=\sqrt{\frac{\sum_{i=1}^{n}{\left(X_{obs}-Y_{i}\right)}^{2}}{n},}$$ (1)

where *D* represents the mean relative error, $Y_{i}$ represents the known SiO_2_ content of weathered or unweathered samples at the sampling point, $X_{obs}$ represents the SiO_2_ content of a particular sampling point of an observed surface of an unweathered sample, and *n* represents the number of samples within the weathered or unweathered set at the sampling point. The outliers are obtained from this process, and the outliers are corrected to the actual amount of weathering.

### 5.2 Weathering in relation to glass type, texture, and color

**FIGURE 3:**
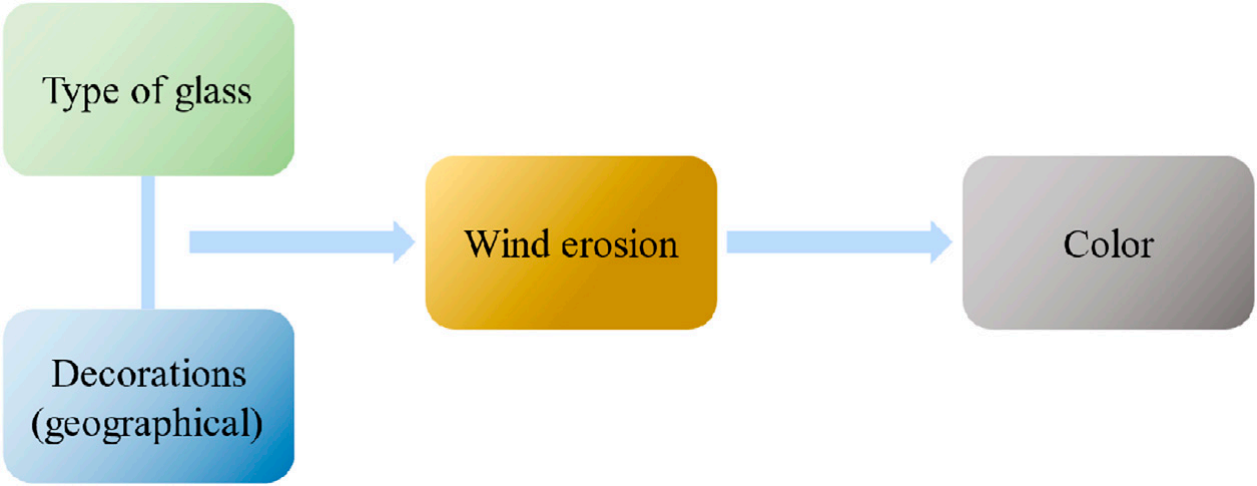
Graph of weathering in relation to glass type, ornamentation and color.

After consulting relevant information, it is found that the degree of weathering of glass is affected by both the type of glass itself and other external factors, such as the burial environment. An important result of weathering is the change of glass color: some areas of glass that were originally green may become purple or gray-yellow. The decoration of the glass does not affect weathering. However, there is a close correspondence between different types of decoration and weathering results, which is contrary to the known theory that ornamentation has little effect on weathering. Therefore, this paper speculates that the decoration of glass, as a symbol of regional culture, represents the geographical environment of different regions, and the natural environment of different regions is an important factor influencing the weathering results. A graph of the relationship between weathering and glass type, decoration, and color is shown in Figure 3.

The glass type and decoration were first analyzed in relation to weathering, and the statistical results are shown in Table 2.

**TABLE 2:** Statistics on weathering and glass types and ornamentation.

Glass type	Ornamentation	Weathering	Quantities
High-potassium glass	A	Unweathered	6
	B	Weathered	6
	C	Unweathered	6
Lead-barium glass	A	Weathered	11
		Unweathered	5
	C	Weathered	17
		Unweathered	7

Table 2 suggests the following conclusions: three types of ornamentation, A, B, and C, are present in high-potassium glass. Of these, the Type B samples are all weathered, while the Type A and Type C samples are unweathered. In the lead-barium glass, the Type A samples are weathered, and the Type C samples are unweathered.

When analyzing the relationship between glass type and weathering, this article uses ornamentation as a control variable. Because lead-barium glass does not contain samples with Type B ornamentation, this paper analyzes Types A and C. Among them, all of the high-potassium glass is not weathered, while the total weathering rate of the Types A and C lead-barium glass is 70%. Therefore, it is concluded that high-potassium glass is less susceptible to weathering than lead-barium glass.

Analyzing the relationship between ornamentation and weathering began with the analysis of Type B ornamentation. In the given sample, all of the glass with Type B ornamentation was weathered, probably because the geographic environment in which the Type B ornamentation was found made it more susceptible to weathering. Therefore, it is speculated that all glass with Type B motifs would be weathered.

### 5.3 Statistical analysis of chemical composition content

The statistical analysis of the chemical composition content is divided into two parts: high-potassium glass and lead-barium glass.

(1) In high-potassium glass, the means, standard deviations, and standard errors of the mean of each component in the weathered and unweathered cases were calculated, and the variance was tested by an independent sample test.

Hypothesis testing of the variance: Consider the statistical hypothesis $H_{0}:\sigma_{1}^{2}=\sigma_{2}^{2};H_{1}:\sigma_{1}^{2}\neq \sigma_{2}^{2},$ and $\mu_{1},\mu_{2}$ are unknown. This hypothesis can be transformed:

$$H_{0}:\frac{\sigma_{1}^{2}}{\sigma_{2}^{2}}=1,$$ (2)

$$H_{1}:\frac{\sigma_{1}^{2}}{\sigma_{2}^{2}}\neq 1.$$ (3)

Because $S_{X}^{1},S_{X}^{2}$ is the minimum variance unbiased statistic for the variance $\sigma_{1}^{2},\sigma_{2}^{2}$ , respectively, $\frac{S_{X}^{1}}{S_{X}^{2}}$ should be in the neighborhood of 1 in case $H_{0}$ holds, and therefore, the rejection domain of $H_{0}$ can be chosen to be of the form:

$$K_{0}=\left\{\frac{S_{X}^{2}}{S_{Y}^{2}}<c_{1},\text{or }\frac{S_{X}^{2}}{S_{Y}^{2}}>c_{2}\right\},c_{1}<c_{2}$$ (4)

By:

$$P\left(\left(\frac{S_{X}^{2}}{S_{Y}^{2}}<c_{1}\right)\cup \left(\frac{S_{X}^{2}}{S_{Y}^{2}}>c_{2}\right)|H_{0}\text{ is established }\right)=\alpha$$ (5)

Order:

$$P\left(\frac{S_{X}^{2}}{S_{Y}^{2}}<c_{1}\right)=P\left(\frac{S_{X}^{2}}{S_{Y}^{2}}>c_{2}\right)=\frac{\alpha }{2},$$ (6)

$$P\left(\frac{S_{X}^{2}}{S_{Y}^{2}}<F_{\frac{\alpha }{2}}\left(n-1,m-1\right)\right)=P\left(\frac{S_{X}^{2}}{S_{Y}^{2}}>F_{1-\frac{\alpha }{2}}\left(n-1,m-1\right)\right)=\frac{\alpha }{2}.$$ (7)

Get the denial field of $H_{0}$ as follows:

$$\frac{S_{X}^{2}}{S_{Y}^{2}}<F_{\frac{\alpha }{2}}\left(n-1,m-1\right)\text{ or }\frac{S_{X}^{2}}{S_{Y}^{2}}>F_{1-\frac{\alpha }{2}}\left(n-1,m-1\right)$$ (8)

The test revealed that the *p*-value > 0.05 for the differences between the relative amounts of potassium oxide, aluminum oxide, copper oxide, phosphorus pentoxide, and tin oxide in the weathered and unweathered cases. Therefore, the original hypothesis could not be rejected. It was concluded that the relative amount of these five compounds did not change due to weathering.

At this point, comparisons of the means of these five components will be made with equal variances. A hypothesis test is performed on the means of two components, at which point the $\sigma_{1}^{2},\sigma_{2}^{2}$ of the two components is known.

When $H_{0}$ holds, there is as follows:

$$\bar{X}∼N\left(\mu_{1},\frac{\sigma_{1}^{2}}{n}\right),$$ (9)

$$\bar{Y}∼N\left(\mu_{2},\frac{\sigma_{2}^{2}}{m}\right).$$ (10)

So, there is as follows:

$$\bar{X}-\bar{Y}∼N\left(0,\frac{\sigma_{1}^{2}}{n}+\frac{\sigma_{2}^{2}}{m}\right),$$ (11)

$$u=\frac{\bar{X}-\bar{Y}}{\sqrt{\frac{\sigma_{1}^{2}}{n}+\frac{\sigma_{2}^{2}}{m}}}∼N\left(0,1\right),$$ (12)

$$P\left(\frac{\bar{X}-\bar{Y}}{\sqrt{\frac{\sigma_{1}^{2}}{n}+\frac{\sigma_{2}^{2}}{m}}}>u_{1-\frac{\alpha }{2}}\right)=\alpha ,$$ (13)

$$P\left(\left|\bar{X}-\bar{Y}\right|>u_{1-\frac{\alpha }{2}}\sqrt{\frac{\sigma_{1}^{2}}{n}+\frac{\sigma_{2}^{2}}{m}}\right)=\alpha .$$ (14)

In this paper, R software was used to test the results obtained. The test results show that the *p*-value > 0.05 for the different amounts of copper oxide and tin oxide with and without weathering, which does not reject the original hypothesis. That is to say, the amounts of copper oxide and tin oxide with and without weathering are equal. The *p*-value < 0.05 for the different amounts of potassium oxide, alumina, and phosphorus pentoxide, which rejects the original hypothesis, that is to say, the relative amounts of potassium oxide, alumina, and phosphorus pentoxide change with weathering.

This results in unequal variances for the residual components of the weathered and unweathered cases. R software is still utilized to realize the hypothesis test for the comparison of two overall means.

From the test results, it can be seen that the test results show that the *p*-value > 0.05 for the different relative amounts of silicon oxide, sodium oxide, lead oxide, barium oxide, and sulfur dioxide with and without weathering, which does not reject the original hypothesis. That is, the relative amounts of silicon oxide, sodium oxide, lead oxide, barium oxide, and sulfur dioxide do not change with weathering.

The *p*-value < 0.05 for the different amounts of calcium oxide, magnesium oxide, iron oxide, and strontium oxide, which rejects the original hypothesis. That is, the relative amounts of calcium oxide, magnesium oxide, iron oxide, and strontium oxide do not change with weathering. However, the total relative amount of silicon oxide is larger before weathering, and the difference between the silicon oxide content in the weathered and unweathered samples is larger; this paper considers that the mean values are unequal.

Therefore, the following equations can be established to determine the pattern of chemical content with and without weathering.

$$y_{i}=x_{i},i=2,8,9,10,13,14$$ (15)

$$y_{i}=a_{i}x_{i},i=1,3,4,5,6,7,11$$ (16)

$$y_{i}=x_{i}+a_{i},i=12$$ (17)

Eq. 15, Eq. 16, and Eq. 17 express the relationship between the components with and without weathering.

Eq. 15 expresses the relationship between the content of the six constituents, sodium oxide, copper oxide, lead oxide, barium oxide, tin oxide, and sulfur dioxide, with or without weathering when the mean values are equal, that is, the content of the unweathered constituents is equal to the content of the weathered constituents.

Eq. 16 represents the relationship between the content of silica, potassium oxide, calcium oxide, magnesium oxide, alumina, iron oxide, and phosphorus pentoxide with and without weathering when the mean values are not equal, that is, the content of the weathered component multiplied by the coefficient $a_{i}$ equals the content of the non-weathered component, of which the values of $a_{1},a_{3},a_{4},a_{5},a_{6,},a_{7},a_{11}$ are 0.74, 17.98, 6.69, 5.35, 3.36, 6.67, and 4.29, respectively. The vector can be expressed as $\left(a_{1},a_{3},a_{4},a_{5},a_{6,},a_{7},a_{11}\right)=\left(0.74,17.98,6.69,5.35,3,36,6.67,4.29\right)$ .

Eq. 17 expresses the relationship between strontium oxide content with and without weathering when the mean values are unequal, that is, the content of the weathered component multiplied by the coefficient $a_{i}$ is equal to the content of the non-weathered component, where the value of $a_{12}$ is 0.03, which can be expressed by the vector $\left(a_{12}\right)=0.03$ .

(2) In lead-barium glass, the mean, standard deviation, and standard error of the mean were calculated for each component in the weathered and unweathered cases, and the independent sample test was conducted to determine the variance. The test revealed that the *p*-values < 0.05 for the difference in the relative amounts of sodium oxide and phosphorus pentoxide with and without weathering. The original hypothesis was rejected. The relative amounts of sodium oxide and phosphorus pentoxide change with weathering. The other components of lead-barium glass did not change with weathering.

Sodium oxide and phosphorus pentoxide have unequal variances with and without weathering, in which case the hypothesis test for the comparison of the two overall means is carried out. From the test results, the *p*-value > 0.05 for the amounts of sodium oxide with and without weathering, which does not reject the original hypothesis that the means with and without weathering are equal. The *p*-value < 0.05 for the relative amounts of phosphorus pentoxide with and without weathering, which rejects the original hypothesis.

The variance of other elements in the weathered and unweathered cases are equal, and the hypothesis test of comparing the two overall means is still carried out. From the test results, it can be seen that the *p*-value > 0.05 for nine components (potassium oxide, magnesium oxide, aluminum oxide, iron oxide, copper oxide, barium oxide, strontium oxide, tin oxide, and sulfur dioxide), which does not reject the original hypothesis, that is, the means with and without weathering are equal. The *p*-value < 0.05 for the amounts of silicon dioxide, calcium oxide, and lead oxide with and without weathering, and the hypothesis is rejected. The relative amounts of silicon dioxide, calcium oxide, and lead oxide change with weathering. The following equation was established to determine the chemical content pattern with and without weathering.

$$y_{i}=x_{i},i=2,3,5,6,7,8,10,12,13,14$$ (18)

$$y_{i}=b_{i}x_{i},i=1,4,9,11$$ (19)

Eq. 18 expresses the relationship between the relative amounts of nine constituents (sodium oxide, potassium oxide, magnesium oxide, aluminum oxide, iron oxide, copper oxide, barium oxide, strontium oxide, tin oxide, and sulfur dioxide), with or without weathering when the mean values are equal, that is, the relative amounts of the unweathered constituents is equal to the relative amounts of the weathered constituents.

Eq. 19 expresses the relationship between the relative amounts of silica, calcium oxide, lead oxide, and phosphorus pentoxide with and without weathering when the mean values are not equal, that is, the content of the weathered component multiplied by the factor $a_{i}$ equals the content of the unweathered component, where $\left(b_{1},b_{4},b_{9},b_{11}\right)=\left(1.97,0.38,0.51,0.20\right)$ .

## 6 Prediction of chemical content of artifacts prior to weathering

### 6.1 Selection and establishment of predictive models

**FIGURE 4:**
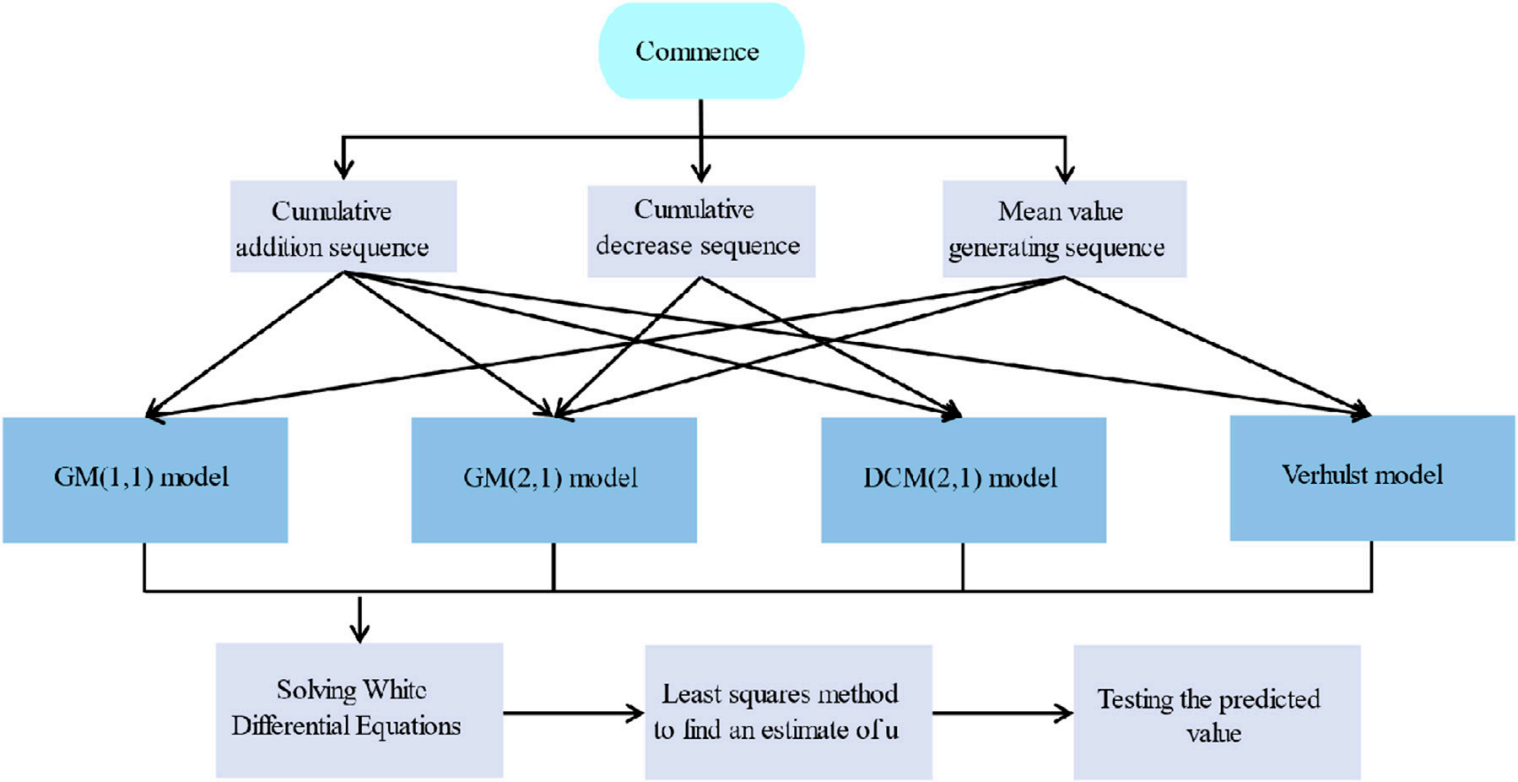
Gray prediction flowchart.

Due to the small amount of sample data explored in this article, a gray prediction model was used to predict the chemical composition content of artifacts before weathering. Gray prediction is a method for predicting systems that contain uncertainties. Gray prediction analyzes the degree of correlation between the various systems and the original data and does a cumulative generation of the sequence and the mean of the sequence to generate the sequence, resulting in a strong regularity of the data sequence. Then, gray prediction establishes a corresponding differential equation model to predict the status of future development trends. Its solving steps are shown in Figure 4.

#### 6.1.1 Examination and processing of data

In order to ensure the feasibility of the modeling method, it is necessary to do the necessary test processing of the “known” data columns. For the high-potassium glass category, the statistical laws already determined show that the content of the four chemical components, silicon dioxide, potassium oxide, calcium oxide, and alumina, changed significantly after weathering. Only these four chemical components are considered in the data predictions. Taking silica as an example, the chemical component content composition sequence of silica for high-potassium glass without weathering is as follows:

$$x^{\left(0\right)}=\left(x^{\left(0\right)}\left(1\right),x^{\left(0\right)}\left(2\right),\ldots x^{\left(0\right)}\left(12\right)\right)$$ (20)

Immediately after that, the level ratio of the sequence is calculated:

$$\lambda \left(k\right)=\frac{x^{\left(0\right)}\left(k-1\right)}{x^{\left(0\right)}\left(k\right)},k=2,3,\ldots ,12.$$ (21)

Then, the sequence can be used as data for the gray prediction model, and all the level ratios fall to satisfy the requirement of tolerable coverage:

$$\theta =\left(e^{-\frac{2}{13}},e^{-\frac{2}{14}}\right).$$ (22)

If it does not satisfy this condition, the sequence *x(0)* needs to be processed with the necessary transformations to make it fall within the admissible cover. In other words, take the appropriate constant *c* and perform a translation transformation:

$$y^{\left(0\right)}\left(k\right)=x^{\left(0\right)}\left(k\right)+c,k=2,3,\ldots ,12$$ (23)

such that the sequence has a grade ratio of

$$\lambda_{y}\left(k\right)=\frac{y^{\left(0\right)}\left(k-1\right)}{y^{\left(0\right)}\left(k\right)}\in \theta ,k=2,3,\ldots ,12.$$ (24)

#### 6.1.2 Modeling

The data column $x^{\left(0\right)}=\left(x^{\left(0\right)}\left(1\right),x^{\left(0\right)}\left(2\right),\ldots x^{\left(0\right)}\left(12\right)\right)$ is obtained by accumulating the generated sequences one at a time:

$$\begin{array}{l} x^{\left(1\right)}=\left(x^{\left(1\right)}\left(1\right),x^{\left(1\right)}\left(2\right),\ldots x^{\left(1\right)}\left(12\right)\right)\\ =\left(x^{\left(0\right)}\left(1\right),x^{\left(0\right)}\left(1\right)+x^{\left(0\right)}\left(2\right),\ldots x^{\left(0\right)}\left(1\right)+\ldots +x^{\left(0\right)}\left(12\right)\right). \end{array}$$ (25)

The mean value of $x^{\left(1\right)}$ generates the sequence $z^{\left(1\right)}=\left(z^{\left(1\right)}\left(2\right),z^{\left(1\right)}\left(3\right),\ldots z^{\left(1\right)}\left(12\right)\right)$ , where:

$$z^{\left(1\right)}\left(k\right)=0.5x^{\left(1\right)}\left(k\right)+0.5x^{\left(1\right)}\left(k-1\right),k=2,3,\ldots ,12.$$ (26)

Subsequently, the gray differential equation is established:

$$x^{\left(0\right)}\left(k\right)+az^{\left(1\right)}\left(k\right),k=2,3,\ldots ,12.$$ (27)

The three matrices of u,Y,B are established, and the corresponding whitened differential equations are as follows:

$$\frac{dx^{\left(1\right)}}{dt}+ax^{\left(1\right)}\left(t\right)=b,$$ (28)

$$u={\left[a,b\right]}^{T},$$ (29)

$$Y={\left[x^{\left(0\right)}\left(2\right),x^{\left(0\right)}\left(3\right),\ldots x^{\left(0\right)}\left(12\right)\right]}^{T},$$ (30)

$$B=\left(\begin{array}{cc} z^{\left(1\right)}\left(2\right) & 1\\ z^{\left(1\right)}\left(3\right) & 1\\ \vdots & \vdots \\ z^{\left(1\right)}\left(12\right) & 1 \end{array}\right).$$ (31)

Then, by least squares, find the make:

$$J\left(u\right)={\left(Y-Bu\right)}^{T}\left(Y-Bu\right).$$ (32)

The estimate of *μ* that reaches the minimum value is as follows:

$$\hat{u}={\left[\hat{a},\hat{b}\right]}^{T}={\left(B^{T}B\right)}^{-1}B^{T}Y.$$ (33)

From there, solving the whitening differential equation yields the predicted values:

$${\hat{x}}^{\left(1\right)}\left(k+1\right)=\left(x^{\left(0\right)}\left(1\right)-\frac{\hat{b}}{\hat{a}}\right)e^{-\hat{a}k}+\frac{\hat{b}}{\hat{a}},k=0,1,\cdots$$ (34)

$${\hat{x}}^{\left(0\right)}\left(k+1\right)={\hat{x}}^{\left(1\right)}\left(k+1\right)-{\hat{x}}^{\left(1\right)}\left(k\right),k=0,1,\ldots$$ (35)

#### 6.1.3 Testing the predicted value

Residuals:

$$\varepsilon \left(k\right)=\frac{x^{\left(0\right)}\left(k\right)-{\hat{x}}^{\left(0\right)}\left(k\right)}{x^{\left(0\right)}\left(k\right)},k=1,2,3,\ldots ,12$$ (36)

In the above equation, $x^{\left(0\right)}\left(1\right)={\hat{x}}^{\left(1\right)}\left(0\right).$ If $\varepsilon \left(k\right)<0.2$ , it is considered to meet the general requirements; if $\varepsilon \left(k\right)<0.1$ , it is considered to meet the higher requirements.

#### 6.1.4 Forecasting

The predicted values of the artifacts before weathering are obtained from the *GM*(1,1) model, and the corresponding predictive forecasts are given according to the actual situation.

### 6.2 Solution of the prediction model

After solving, it can be concluded that the data series of the silica content of high-potassium glass is as follows:

$$\begin{array}{l} x^{\left(0\right)}=\left(x^{\left(0\right)}\left(1\right),x^{\left(0\right)}\left(2\right),\ldots x^{\left(0\right)}\left(12\right)\right)\\ =\left(69.33,87.05,61.71,65.88,61.58,67.65,59.81,62.47,65.18,79.46,76.68\right) \end{array}$$ (37)

Its class ratio is as follows:

$$\lambda \left(k\right)=\left(0.7964,1.4106,0.9367,1.0698,0.9103,1.1311,1.0136,0.9446,0.9584,0.8203,1.0363\right).$$ (38)

Because all the $\lambda \left(k\right)\in \left[0.7964,1.4106\right],k=2,3,\cdots ,12$ , this can be used as the model of the sequence of data for gray prediction; the original data at a time cumulative obtained:

$$x^{\left(1\right)}=\left(69.33,156.38,218.09,283.97,345.55,413.20,473.01,532.02,594.49659.67,739.13,815.81\right)$$ (39)

Construct data matrices and data vectors:

$$B=\left(\begin{array}{cc} -112.855 & 1\\ -187.235 & 1\\ -251.03 & 1\\ -314.76 & 1\\ -379.375 & 1\\ -443.105 & 1\\ -502.515 & 1\\ -563.255 & 1\\ -627.08 & 1\\ -699.4 & 1\\ -777.47 & 1 \end{array}\right)$$ (40)

$$Y=\left[\begin{array}{c} 87.05\\ 61.71\\ 65.88\\ 61.58\\ 67.65\\ 59.81\\ 59.01\\ 62.47\\ 65.18\\ 79.46\\ 76.68 \end{array}\right]$$ (41)

Calculated through the matrix:

$$\hat{u}={\left[\hat{a},\hat{b}\right]}^{T}={\left(B^{T}B\right)}^{-1}B^{T}Y=\left[\begin{array}{c} -0.0015\\ 67.1997 \end{array}\right]$$ (42)

This can be obtained from $\hat{a}=-0.0015,\hat{b}=67.1997$ . Thus, the differential equation is established:

$$\frac{dx^{\left(1\right)}\left(t\right)}{dt}+ax^{\left(1\right)}\left(t\right)=b$$ (43)

Eventually available:

$$\begin{array}{l} {\hat{x}}^{\left(1\right)}\left(k+1\right)\\ =\left(x^{\left(0\right)}\left(1\right)-\frac{\hat{b}}{\hat{a}}\right)e^{-\hat{a}k}+\frac{\hat{b}}{\hat{a}}\\ =44869.13e^{0.0015k}-44799.8 \end{array}$$ (44)

**FIGURE 5:**
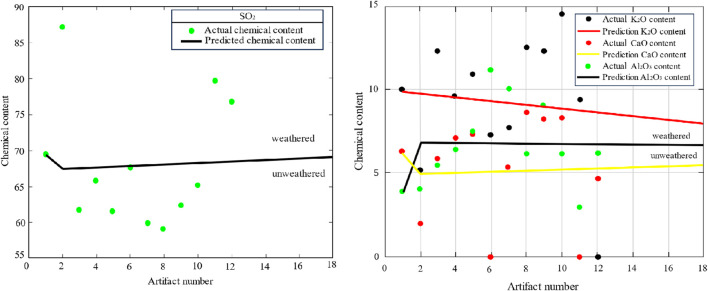
Lead-barium major element projections.

Representative chemical components of high-potassium glass and lead-barium glass, such as silica, potassium oxide, and lead oxide, are selected for analysis. The predicted content map of the main elements in the high-potassium category is shown in Figure 5.

**FIGURE 6:**
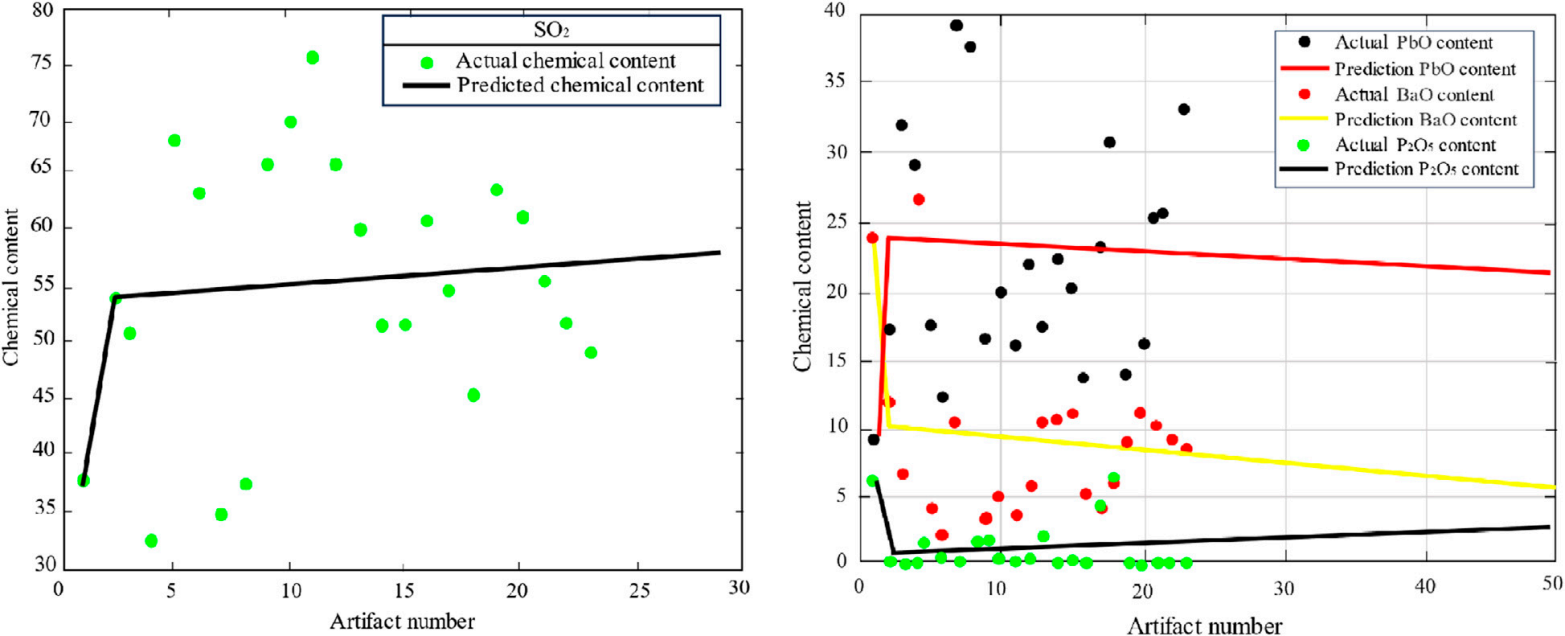
Lead-barium major element projections.

The data analysis was carried out by MATLAB, and it can be seen from Figure 5 that the predicted silica content of weathered high-potassium glass increases continuously with the amount of weathering, while the relative amounts of potassium oxide and calcium oxide decrease. The silica content of unweathered high-potassium glass varies. The prediction of the main components of the lead-barium glass is shown in Figure 6.

Figure 6 shows that the predicted values of silica content in lead-barium glass before weathering are 57.534%, 58.171%, and 60.787% for the three severe weathering sample points of 08, 26, and 54, respectively. That is to say, the silica content decreases while the percentages of lead oxide and barium oxide increase with increased weathering.

The results of the calculations are shown in Table 3 and Table 4 as an example. Due to space limitations in the main text, only some of the detailed data are shown.

**TABLE 3:** Predicted chemical composition content of glass before weathering.

ASP	SiO_2_	Na_2_O	K_2_O	CaO	MgO	Al_2_O_3_	Fe_2_O _3_
11	25.1	0.0	0.0	1.2	0.0	1.9	0.0
25 UP	25.1	0.0	0.0	1.2	0.0	1.9	0.0
34	33.6	0.0	0.0	1.2	0.0	1.9	0.0
36	33.6	0.0	0.0	1.2	0.0	1.9	0.0
37	67.8	2.1	14.5	8.3	0.5	6.2	0.4
38	33.6	0.0	0.0	1.2	0.0	1.9	0.0

*Where ASP is artifact sampling points, SiO_2_ is silicon dioxide, Na_2_O is sodium oxide, K_2_O is potassium oxide, CaO is calcium oxide, MgO is magnesium oxide, Al_2_O_3_ is aluminum oxide, Fe_2_O_3_ is iron oxide, and UP is unweathered points.

**TABLE 4:** Predicted chemical composition content of weathered high-potassium glass before weathering.

ASP	CuO	PbO	BaO	P_2_O_5_	SrO	SnO_2_	SO_2_
11	0.8	41.3	15.5	2.5	0.0	0.0	0.0
25 UP	0.8	41.3	15.5	2.5	0.0	0.0	0.0
34	0.8	41.3	15.5	2.5	0.0	0.0	0.0
36	0.8	41.3	15.5	2.5	0.0	0.0	0.0
37	1.1	0.1	0.0	0.0	0.0	0.0	0.0
38	0.8	41.3	15.5	2.5	0.0	0.0	0.0

*Where ASP is artifact sampling points, CuO is copper oxide, PbO is lead oxide, BaO is barium oxide, P_2_O_5_ is phosphorus pentoxide, SrO is strontium oxide, SnO_2_ is tin oxide, SO_2_ is sulfur dioxide, and UP is unweathered points.

## 7 Exploring differences in chemical constituent associative relationships

### 7.1 Disparity modeling

This article will further analyze the correlations between chemical compositions for different categories of glass artifact samples and compare the differences in the correlations between different categories.

In this article, the generalized Shapley function based on a better is selected to analyze the correlation relationship between indicators, where λ denotes the fuzzy measure that can analyze the interactions between individual indicators relative to other remaining arbitrary indicator sets and is also able to analyze the interactions between arbitrary indicator sets relative to the remaining arbitrary indicator sets. This fuzzy measure can make the analysis results more accurate and more in line with the reality.

The use of this analysis requires data standardization, and when the indicator $C_{j}$ is numerical, the data are processed and standardized to obtain it:

$$y_{ij}=\frac{a_{ij}}{\sqrt{\sum_{i=1}^{n}{\left(a_{ij}\right)}^{2}}}$$ (45)

Let $X=\left\{x_{1},x_{2},\ldots ,x_{n}\right\}$ be a nonempty set and *P(X)* be a power set on *X* $\lambda \in \left(-1,\infty \right)$ , $\mu :P\left(X\right)\to \left[0,1\right]$ , if *P(X)* satisfies the following three points: (i) $\mu \left(0\right)=0,\mu \left(X\right)=1;$ ② $\forall A,B\in P\left(X\right),A\subset B,$ then $\mu \left(A\right)\leq \mu \left(B\right);$ ③ $\mu \left(A\cup B\right)=\mu \left(A\right)+\mu \left(B\right)+\lambda \mu \left(A\right)\lambda \mu \left(B\right)$ , then μ is said to be the λ - fuzzy measure on *X* can be found: ① When λ=0 , which can be obtained from $\mu \left(A\cup B\right)=\mu \left(A\right)+\mu \left(B\right)$ , then A and B are independent of each other; ② When λ<0 , $\mu \left(A\cup B\right)<\mu \left(A\right)+\mu \left(B\right)$ , then A and B have a cross-relationship; ③ When λ>0 , $\mu \left(A\cup B\right)>\mu \left(A\right)+\mu \left(B\right)$ , then A and B have a complementary relationship.

Based on the correlation relationship between indicators of λ - fuzzy measure, let $\mu \left(x_{i}\right)$ denote the fuzzy measure. Then, the fuzzy measure formula of A is as follows:

$$\mu \left(A\right)=\left\{\begin{array}{ll} \frac{1}{\lambda }\left(\prod_{x_{j}\in A}\left[1+\lambda \mu \left(x_{j}\right)-1\right]\right), & \lambda \neq 0\\ \sum_{x_{j}\in A}\mu \left(x_{j}\right), & \lambda =0 \end{array}\right.$$ (46)

Let *A = X*, then $\mu \left(A\right)=\mu \left(X\right)=1$ can be obtained:

$$\lambda +1=\prod_{j=1}^{m}\left[1+\lambda \mu \left(x_{j}\right)\right],-1<\lambda <\infty ,\lambda \neq 0$$ (47)

The Shapley function formula based on the λ - fuzzy measure is as follows:

$$g_{s}\left(g,X\right)=\sum_{T\subseteq \left(X-S\right)}\frac{\left(n-s-t\right)!t!}{\left(n-s+1\right)!}\left[\mu \left(A\cup T\right)-\mu \left(T\right)\right]$$ (48)

where *X* denotes the full set of indicators, *S* denotes a subset in *X*, *X-S* is the difference set between the set *X* and the set *S*, *T* denotes a subset in *X-S*, and *n, T, S* denotes the base of *X, T, S*.

### 7.2 Differential modeling solutions for exploring chemical compositional correlations

The above study makes it clear that studying the relationship between the chemical composition of different glass artifact samples requires consideration of weathering. Hence, we address four categories.

(1) High-potassium glass in a weathered background

First, for weathered high-potassium glass, the standard values of individual chemical components are shown in Table 5, and the importance of each component is shown in Table 6. The calculated fuzzy measurements are shown in Table 7.

**TABLE 5:** Standard values of each component of high-potassium glass in the context of weathering.

Chemical composition	SiO_2_	Na_2_O	KO_2_	CaO	MgO	Al_2_O_3_	Fe_2_O_3_
Standard value	0.9	0	0.0	0.0	0.0	0.0	0.0
Chemical composition	CuO	PbO	BaO	P O_25_	SrO	SnO_2_	SO_2_
Standard value	0.0	0	0	0.0	0	0	0

**TABLE 6:** Importance of each component of high-potassium glass in the context of weathering.

Entropy weight	$\mu \left(C_{1}\right)$	$\mu \left(C_{2}\right)$	$\mu \left(C_{3}\right)$	$\mu \left(C_{4}\right)$	$\mu \left(C_{5}\right)$	$\mu \left(C_{6}\right)$	$\mu \left(C_{7}\right)$
Significance	0.1	0	0.2	0.1	0.1	0.1	0.2
Entropy weight	$\mu \left(C_{8}\right)$	$\mu \left(C_{9}\right)$	$\mu \left(C_{10}\right)$	$\mu \left(C_{11}\right)$	$\mu \left(C_{12}\right)$	$\mu \left(C_{13}\right)$	$\mu \left(C_{14}\right)$
Significance	0.1	0	0	0.1	0	0	0

**TABLE 7:** Fuzzy measures of the chemical composition of high-potassium glasses in the context of weathering.

Fuzzy measure	Fuzzy measurements	Fuzzy measure	Fuzzy measurements	Fuzzy measure	Fuzzy measurements
$\mu \left(C_{1},C_{3}\right)$	0.3	$\mu \left(C_{3},C_{8}\right)$	0.3	$\mu \left(C_{1},C_{3},C_{9}\right)$	0.2
$\mu \left(C_{1},C_{4}\right)$	0.5	$\mu \left(C_{3},C_{9}\right)$	0	$\mu \left(C_{1},C_{4},C_{9}\right)$	0.2
$\mu \left(C_{1},C_{8}\right)$	0.4	$\mu \left(C_{3},C_{10}\right)$	0	$\mu \left(C_{1},C_{9},C_{10}\right)$	0.2
$\mu \left(C_{1},C_{9}\right)$	0	$\mu \left(C_{3},C_{11}\right)$	0	$\mu \left(C_{3},C_{9},C_{10}\right)$	0.1
$\mu \left(C_{1},C_{10}\right)$	0	$\mu \left(C_{9},C_{10}\right)$	0	$\mu \left(C_{3},C_{9},C_{11}\right)$	0.2
$\mu \left(C_{1},C_{11}\right)$	0.3	$\mu \left(C_{9},C_{11}\right)$	0	$\mu \left(C_{1},C_{3},C_{9},C_{10}\right)$	0.1

According to the fuzzy measurements in Table 12, the chemical composition of weathered high-potassium glass is not highly correlated, and its correlation is weak.

(2) Lead-barium glass in a weathered context

For weathered lead-barium glass, the standard values of the individual chemical compositions are shown in Table 8. The resulting calculated chemical compositional importance is shown in Table 9. The calculated fuzzy measurements are shown in Table 10.

**TABLE 8:** Standard values for each component of lead-barium glass in the weathering background.

Chemical composition	SiO_2_	Na_2_O	KO_2_	CaO	MgO	Al_2_O_3_	Fe_2_O_3_
Standard value	0.3	0.0	0.0	0.0	0.0	0.0	0.0
Chemical composition	CuO	PbO	BaO	P O_25_	SrO	SnO_2_	SO_2_
Standard value	0.0	0.4	0.1	0.0	0.0	0.1	0.0

**TABLE 9:** Importance of each component of lead-barium glass in the context of weathering.

Entropy weight	$\mu \left(C_{1}\right)$	$\mu \left(C_{2}\right)$	$\mu \left(C_{3}\right)$	$\mu \left(C_{4}\right)$	$\mu \left(C_{5}\right)$	$\mu \left(C_{6}\right)$	$\mu \left(C_{7}\right)$
Significance	0.1	0.1	0.0	0.1	0.1	0.1	0.1
Entropy weight	$\mu \left(C_{8}\right)$	$\mu \left(C_{9}\right)$	$\mu \left(C_{10}\right)$	$\mu \left(C_{11}\right)$	$\mu \left(C_{12}\right)$	$\mu \left(C_{13}\right)$	$\mu \left(C_{14}\right)$
Significance	0.1	0.1	0.1	0.1	0.1	0.0	0.0

**TABLE 10:** Degree of correlation between the components of lead-barium glass in the context of weathering.

Fuzzy measure	Fuzzy measurements	Fuzzy measure	Fuzzy measurements	Fuzzy measure	Fuzzy measurements
$\mu \left(C_{1},C_{3}\right)$	0.4	$\mu \left(C_{3},C_{8}\right)$	0.3	$\mu \left(C_{1},C_{3},C_{9}\right)$	0.5
$\mu \left(C_{1},C_{4}\right)$	0.4	$\mu \left(C_{3},C_{9}\right)$	0.4	$\mu \left(C_{1},C_{4},C_{9}\right)$	0.5
$\mu \left(C_{1},C_{8}\right)$	0.3	$\mu \left(C_{3},C_{10}\right)$	0.4	$\mu \left(C_{1},C_{9},C_{10}\right)$	0.6
$\mu \left(C_{1},C_{9}\right)$	0.5	$\mu \left(C_{3},C_{11}\right)$	0.4	$\mu \left(C_{3},C_{9},C_{10}\right)$	0.5
$\mu \left(C_{1},C_{10}\right)$	0.6	$\mu \left(C_{9},C_{10}\right)$	0.5	$\mu \left(C_{3},C_{9},C_{11}\right)$	0.5
$\mu \left(C_{1},C_{11}\right)$	0.4	$\mu \left(C_{9},C_{11}\right)$	0.5	$\mu \left(C_{1},C_{3},C_{9},C_{10}\right)$	0.6

According to the fuzzy measure, there is a medium degree of correlation for the chemical composition of weathered lead-barium glass: there is a correlation between the chemical content of silica, barium oxide, and lead oxide.

(3) High-potassium glass in an unweathered background

For unweathered high-potassium glass, the standard values for individual chemical compositions are shown in Table 11. The resulting calculated chemical compositional importance is shown in Table 12. The calculated fuzzy measurements are detailed in Table 13.

**TABLE 11:** Degree of correlation of the components of high-potassium glass in the context of no weathering.

Chemical composition	SiO_2_	Na_2_O	KO_2_	CaO	MgO	Al_2_O_3_	Fe_2_O_3_
Standard value	0.7	0.0	0.1	0.1	0.0	0.1	0.0
Chemical composition	CuO	PbO	BaO	P O_25_	SrO	SnO_2_	SO_2_
Standard value	0.0	0.0	0.0	0.0	0.0	0.0	0.0

**TABLE 12:** Importance of each component of high-potassium glass in the context of no weathering.

Entropy weight	$\mu \left(C_{1}\right)$	$\mu \left(C_{2}\right)$	$\mu \left(C_{3}\right)$	$\mu \left(C_{4}\right)$	$\mu \left(C_{5}\right)$	$\mu \left(C_{6}\right)$	$\mu \left(C_{7}\right)$
Significance	0.1	0.1	0.1	0.1	0.1	0.1	0.0
Entropy weight	$\mu \left(C_{8}\right)$	$\mu \left(C_{9}\right)$	$\mu \left(C_{10}\right)$	$\mu \left(C_{11}\right)$	$\mu \left(C_{12}\right)$	$\mu \left(C_{13}\right)$	$\mu \left(C_{14}\right)$
Significance	0.1	0.1	0.0	0.1	0.1	0.0	0.1

**TABLE 13:** Degree of correlation of the components of high-potassium glass in the context of no weathering.

Fuzzy measure	Fuzzy measurements	Fuzzy measure	Fuzzy measurements	Fuzzy measure	Fuzzy measurements
$\mu \left(C_{1},C_{3}\right)$	0.7	$\mu \left(C_{3},C_{8}\right)$	0.4	$\mu \left(C_{1},C_{3},C_{9}\right)$	0.4
$\mu \left(C_{1},C_{4}\right)$	0.6	$\mu \left(C_{3},C_{9}\right)$	0.3	$\mu \left(C_{1},C_{4},C_{9}\right)$	0.5
$\mu \left(C_{1},C_{8}\right)$	0.5	$\mu \left(C_{3},C_{10}\right)$	0.3	$\mu \left(C_{1},C_{9},C_{10}\right)$	0.2
$\mu \left(C_{1},C_{9}\right)$	0.3	$\mu \left(C_{3},C_{11}\right)$	0.4	$\mu \left(C_{3},C_{9},C_{10}\right)$	0.4
$\mu \left(C_{1},C_{10}\right)$	0.2	$\mu \left(C_{9},C_{10}\right)$	0.5	$\mu \left(C_{3},C_{9},C_{11}\right)$	0.4
$\mu \left(C_{1},C_{11}\right)$	0.4	$\mu \left(C_{9},C_{11}\right)$	0.4	$\mu \left(C_{1},C_{3},C_{9},C_{10}\right)$	0.3

According to the fuzzy measurements in Table 13, it can be seen that the degree of correlation is not high for the chemical composition of unweathered high-potassium glass, but there is a correlation between the chemical content of silica and calcium oxide.

(4) Lead-barium glass in an unweathered background

For unweathered lead-barium glass, the standard values for the individual chemical constituents are shown in Table 14. The resulting calculated chemical compositional importance is shown in Table 15. The calculated fuzzy measurements are shown in Table 16.

**TABLE 14:** Degree of correlation between the components of lead-barium glass in the context of no weathering.

Chemical composition	SiO_2_	Na_2_O	KO_2_	CaO	MgO	Al_2_O_3_	Fe_2_O_3_
Standard value	0.5	0.0	0.0	0.0	0.0	0.0	0.0
Chemical composition	CuO	PbO	BaO	P O_25_	SrO	SnO_2_	SO_2_
Standard value	0.0	0.2	0.1	0.0	0.0	0.0	0.0

**TABLE 15:** Importance of each component of lead-barium glass in the context of no weathering.

Entropy weight	$\mu \left(C_{1}\right)$	$\mu \left(C_{2}\right)$	$\mu \left(C_{3}\right)$	$\mu \left(C_{4}\right)$	$\mu \left(C_{5}\right)$	$\mu \left(C_{6}\right)$	$\mu \left(C_{7}\right)$
Significance	0.1	0.1	0.0	0.1	0.1	0.1	0.1
Entropy weight	$\mu \left(C_{8}\right)$	$\mu \left(C_{9}\right)$	$\mu \left(C_{10}\right)$	$\mu \left(C_{11}\right)$	$\mu \left(C_{12}\right)$	$\mu \left(C_{13}\right)$	$\mu \left(C_{14}\right)$
Significance	0.1	0.1	0.1	0.0	0.1	0.1	0.0

**TABLE 16:** Degree of correlation of the components of lead-barium glass in the context of no weathering.

Fuzzy measure	Fuzzy measurements	Fuzzy measure	Fuzzy measurements	Fuzzy measure	Fuzzy measurements
$\mu \left(C_{1},C_{3}\right)$	0.6	$\mu \left(C_{3},C_{8}\right)$	0.3	$\mu \left(C_{1},C_{3},C_{9}\right)$	0.5
$\mu \left(C_{1},C_{4}\right)$	0.6	$\mu \left(C_{3},C_{9}\right)$	0.4	$\mu \left(C_{1},C_{4},C_{9}\right)$	0.4
$\mu \left(C_{1},C_{8}\right)$	0.6	$\mu \left(C_{3},C_{10}\right)$	0.4	$\mu \left(C_{1},C_{9},C_{10}\right)$	0.5
$\mu \left(C_{1},C_{9}\right)$	0.5	$\mu \left(C_{3},C_{11}\right)$	0.3	$\mu \left(C_{3},C_{9},C_{10}\right)$	0.5
$\mu \left(C_{1},C_{10}\right)$	0.4	$\mu \left(C_{9},C_{10}\right)$	0.5	$\mu \left(C_{3},C_{9},C_{11}\right)$	0.5
$\mu \left(C_{1},C_{11}\right)$	0.4	$\mu \left(C_{9},C_{11}\right)$	0.4	$\mu \left(C_{1},C_{3},C_{9},C_{10}\right)$	0.6

Table 16 shows the degree of correlation for the chemical composition of unweathered lead-barium glass is high, and there is a certain correlation consistency relationship.

The Shapley function values for each category are then calculated according to Eq. 48. For weathered high-potassium glass, $g_{1}\left(g,X\right)=0.305$ ; for weathered lead-barium glass, $g_{2}\left(g,X\right)=0.431$ , for unweathered high-potassium glass, $g_{3}\left(g,X\right)=0.394$ , and for unweathered lead-barium glass, $g_{4}\left(g,X\right)=0.497$ .

It is clear that there is a difference between these correlations. The Shapley function values progressively decrease, and the correlations become weaker from unweathered lead-barium glass to weathered lead-barium glass to unweathered high-potassium glass and finally to weathered high-potassium glass.

## 8 Conclusion

This paper focuses on the statistical analysis, prediction, and correlation analysis of the chemical and molecular composition of ancient glassware.

R software is used to realize the hypothesis test for the comparison of two overall means. Equations are established to determine the pattern of the chemical content of different types of glass with and without weathering. A gray prediction model was used to predict the chemical content of artifacts before weathering. Data analysis was carried out through MATLAB. With increased weathering degree, the relative content of silica gradually decreases while the content of both lead oxide and barium oxide increases. The generalized Shapley function based on fuzzy measurements is selected to analyze the correlation relationship between indicators, to analyze the correlation relationship between their chemical compositions, and to compare the differences in the correlation relationship of chemical compositions between different categories. It can be clearly seen that there are differences between these correlations. The Shapley function values gradually decrease, and the correlations become weaker from unweathered lead-barium glass to weathered lead-barium glass to unweathered high-potassium glass and finally to weathered high-potassium glass. The scheme proposed in this paper can solve the difficult problem of category judgment in archeology, which is of great significance in promoting the smooth progress of archaeological work.

## Data Availability

The original contributions presented in the study are included in the article/Supplementary material; further inquiries can be directed to the corresponding author.
